# Structural Characterization and Antioxidant Activity of Exopolysaccharide Produced from Beet Waste Residue by *Leuconostoc pseudomesenteroides*

**DOI:** 10.3390/antiox13111289

**Published:** 2024-10-25

**Authors:** Ying Liu, Ying Zhou, Cong Bian, Heqi Li, Youxian Kang, Yu Gao, Yao Peng, Chunjing Zhang

**Affiliations:** 1 Department of Biochemistry and Molecular Biology, Qiqihar Medical University, Qiqihar 161000, China; 001256@qmu.edu.cn (Y.L.); 2022210123@stu.qmu.edu.cn (Y.Z.); 2022210107@stu.qmu.edu.cn (C.B.); 2023210118@stu.qmu.edu.cn (H.L.); 2023210107@stu.qmu.edu.cn (Y.K.); 864555482@qmu.edu.cn (Y.P.); 2 Department of Clinical Trial, The Third Affiliated Hospital of Qiqihar Medical University, Qiqihar 161000, China; gaoyu6212@qmu.edu.cn

**Keywords:** exopolysaccharides, *Leuconostoc pseudomesenteroides*, beet waste residue, structure and characteristics, antioxidant activity

## Abstract

Lactic acid bacteria exopolysaccharide (EPS) is a large molecular polymer produced during the growth and metabolism of lactic acid bacteria. EPS has multiple biological functions and is widely used in fields such as food and medicine. However, the low yield and high production cost of EPS derived from lactic acid bacteria limit its widespread application. In this study, we used beet waste residue as a substrate to produce EPS by fermentation with *Leuconostoc pseudomesenteroides* to improve the utilization rate of agricultural waste and reduce the production cost of lactic acid bacterial EPS. After purification, the molecular weight (Mw) of EPS was determined to be 417 kDa using high-performance size exclusion chromatography (HPSEC). High-performance liquid chromatography (HPLC), Fourier transform infrared (FTIR) spectroscopy, and nuclear magnetic resonance (NMR) spectroscopy revealed that the EPS was composed of glucose subunits with α-1,6 glycosidic linkages. The thermal analysis and heavy metal adsorption capacity revealed a relatively high degradation temperature of 315.54 °C and that the material could effectively adsorb Cu^2+^. Additionally, the findings indicated that the EPS exhibited a significant ability to neutralize free radicals, a property that was found to be concentration dependent. Furthermore, the results of the intracellular study showed the protective effect of freshly isolated EPS on tBHP-induced cellular oxidative stress at a concentration of 50 µg/mL. These results suggest that the EPS from *L. pseudomesenteroides* may be developed as antioxidant agents for functional food products and pharmaceutical applications due to its capacity to scavenge free radicals.

## 1. Introduction

Microbial polysaccharides are important and complex polymers that include exopolysaccharides, capsular polysaccharides, and lipopolysaccharides [1]. EPSs are secondary metabolites that can be secreted by bacteria. Among the bacteria studied, EPSs synthesized by some lactic acid bacteria have a significant advantage as they are recognized as probiotics and have been granted GRAS (generally regarded as safe) status by the WHO [2]. In recent years, an increasing number of studies have focused on the characteristics and functionalities of EPSs synthesized by lactic acid bacteria. This focus is primarily due to their unique molecular configurations, functional properties, and manufacturing processes, which have attracted significant attention in academic research and industry [3,4,5]. Lactic acid bacteria EPSs can be classified into homopolysaccharides and heteropolysaccharides based on the monomeric composition of the polysaccharide chains. Homopolysaccharides are composed of a single type of sugar monomer and are named based on the monosaccharide type and the anomeric configuration between individual units [6]. For example, homopolysaccharides derived from glucose can be categorized as α-glucans or β-glucans. Heteropolysaccharides contain two or more different types of monosaccharides, and the specific arrangement and combination of these monosaccharides in heteropolysaccharides give rise to diverse structures [7]. Notably, EPSs synthesized by lactic acid bacteria, particularly bacteria within the *Leuconostoc* and *Weissella* genera, predominantly feature α-glucans with a linear structure [8,9]. The production of EPSs by lactic acid bacteria has relied predominantly on commercial sucrose as a carbon substrate. Sucrose, which is commonly obtained from edible fruits and vegetables, is known for its significant nutritional benefits to humans [10,11]. Improvements in EPS production through affordable and environmentally sustainable methods are urgently needed to meet the growing market demand. Therefore, the focus of researchers has shifted toward the use of cost-effective alternatives such as agricultural waste to economize the synthesis of EPSs from chemical precursors. In recent years, the production of EPSs from ecofriendly carbon sources instead of sucrose has attracted extensive attention [3,12].

*Beta vulgaris* subsp., commonly known as sugar beet, is highly important in the agricultural sector because of its nutritional value and versatility in industrial applications. It primarily serves as a key ingredient for sucrose manufacturing, accounting for approximately 16% of the world’s sugar output [13]. In 2017, global sugar beet production reached approximately 300 million tons, with Europe playing a dominant role and contributing approximately 70% [14]. The conventional method of processing sugar beet employs hot water to extract sucrose. Following this extraction step, the resulting juice undergoes purification, concentration, and crystallization to yield refined sugar. The beet waste residue is rich in sucrose, cellulose, hemicelluloses, and pectin; it either serves as animal feed or acts as a base material in the production of biogas [15]. Therefore, the use of beet waste residue to produce EPSs can not only reduce the cost of sugar production, but also realize the efficient use of resources. However, many studies have used agricultural waste to replace sucrose in the medium and optimize the composition and culture conditions of the medium, and few studies have directly fermented agricultural waste with lactic acid bacteria to produce EPSs. Lactic acid bacterial fermentation technology has been utilized for preserving food and enhancing its sensory and functional properties such as antioxidant, hypoglycemic, and lipid-lowering effects [16,17]. Published studies have consistently shown that fermentation plays a significant role in altering the structure and functionality of lactic acid bacteria EPSs.

Oxidative stress, resulting from the excessive intracellular accumulation of reactive oxygen species (ROS) and other free radical species, contributes to the onset progression of various diseases [18]. Oxidative stress has been shown to participate in a wide range of diseases including cardiovascular disease [19], steatotic liver disease [20] as well as diabetes [21], etc., which has revealed the multiple mechanisms by which oxidants contribute to cellular damage [22]. In recent years, natural antioxidants, especially lactic acid bacteria EPSs, have received enormous attention due to their low toxicity and naturalness. Lactic acid bacteria EPSs have been shown to enhance cellular defense mechanisms through antioxidant activity that reduces the oxidative damage caused by ROS and free radicals [23]. In this study, *Leuconostoc pseudomesenteroides* was used to ferment beet waste residue to produce EPS, and the EPS was isolated and purified. Moreover, the structure and properties of the EPS were identified, and its intracellular antioxidant activities explored.

## 2. Materials and Methods

### 2.1. Strains and Production of EPS by Fermentation

The EPS-producing strain *L. pseudomesenteroides* was isolated from homemade wine and stored in MRS broth; the strain was a gift from Professor Du [24]. The remaining beet waste residue (500 g) from sugar frying was sterilized for 15 min at 121 °C and then transferred into a 5 L sterilized glass fermenter. Previous determinations indicated a sugar content of 131.5 g/kg in beet waste residue. Subsequently, 4 L of deionized water was added to the fermenter. Next, 5% *L. pseudomesenteroides* was added, followed by static incubation at 30 °C. Time-course experiments were conducted to investigate EPS production and bacterial growth. The growth curve was determined based on colony-forming units (CFUs), and samples of the fermentation broth were assayed every 12 h over a period of 144 h. The yield of EPS and CFUs were determined. EPS yield was defined as EPS produced/beet waste residue, while the CFU yield was defined as CFU produced/beet waste residue.

### 2.2. Extraction and Purification of EPS

The production and isolation of EPS followed the protocol outlined by Zhao et al. [25]. The cell biomass was removed through centrifugation at 12,000× *g* for 20 min and the supernatant was collected. Protein impurities were eliminated by adding 10% (*w*/*v*) trichloroacetic acid (TCA), followed by another centrifugation step (12,000× *g* for 20 min at 4 °C). Three volumes of cold ethanol were then mixed with the supernatant and precipitated at 4 °C overnight. The resulting precipitate was obtained via centrifugation (12,000× *g* for 30 min) and dissolved in deionized water. A crude EPS suspension was prepared by dialyzing the solution against deionized water using a 12–14 kDa dialysis membrane (Shanghai Yuanye Bio-Technology Co., Ltd., Shanghai, China) at 4 °C for two days. The dialyzed sample was freeze-dried, weighed, and preserved. The sugar concentration in the EPS was determined using the phenol-sulfuric acid method [26]. The EPS sample, with a concentration of 50 mg/mL, was passed through a Sephadex G-100 column (dimensions: 16 mm in diameter × 100 cm in length, Cytiva, Marlborough, MA, USA) and eluted using deionized water at a flow rate of 0.2 mL/min. The fractions containing EPS were identified via the phenol-sulfuric acid method, combined, subjected to dialysis, and finally freeze-dried in preparation for the structural analysis.

### 2.3. Purity, Monosaccharide Composition, and Molecular Weight (Mw) of EPS

The purity of the EPS fraction was assessed using a UV–visible spectrophotometer at wavelengths ranging from 190 to 300 nm (Spectrum Instruments, SP-1920UV, Shanghai, China).

The monosaccharide constituents were analyzed using high-performance liquid chromatography (HPLC, LC-20AT, Shimadzu, Kyoto, Japan), according to the methodology outlined by Yang et al. [6]. A 10 mg portion of EPS was incubated with 2 mL of 2 M trifluoroacetic acid (TFA) at a temperature of 120 °C for a duration of 6 h for digestion. The excess TFA was subsequently eliminated via evaporation. The derived mixture underwent acetylation before the individual monosaccharides were identified by comparing their retention times with those of standard sugar references.

The Mw of the purified EPS was determined using high-performance size exclusion chromatography (HPSEC, DGU-14A, Shimadzu, Kyoto, Japan). The HPSEC system utilized in this study included a Shodex OH-park SB-805 (8.0 mm × 300 mm) column with an RI detector model. The EPS was eluted with ultrapure water at a flow rate of 1 mL/min, an internal temperature of 40 °C, and an injection volume of 10 μL.

### 2.4. FTIR Spectroscopy Analysis of EPS

The major functional groups of EPS were identified using Fourier transform infrared (FTIR) spectroscopy (FTS3000, Bruker, Karlsruhe, Germany). The potassium bromide (KBr) method was employed, where 10 mg of EPS was mixed with dry KBr and then compressed to form a pellet. The EPS KBr pellet was analyzed by measuring the spectrum in the 400–4000 cm^−1^ region at a resolution of 4 cm^−1^ using 32 scans.

### 2.5. X-ray Diffraction (XRD) Analysis of EPS

The phase constitution and crystalline nature of EPS were analyzed using XRD (X’Pert PRO MPD, Panalytical, Almelo, The Netherlands). The examination involved the operation of an XRD apparatus within a 2θ spectrum ranging from 10° to 80°, with increments of 10° in 2θ intervals. The crystallinity index (*CI_xrd_*) of the samples was calculated by the ratio of the peaks of crystalline phases and total areas of crystalline and amorphous peaks according to Equation (1).

(1) $$CI_{xrd}=\frac{\sum A_{crystal}}{\left(\sum A_{crystal}+\sum A_{amorphous}\right)}$$

### 2.6. Analysis of the Carbohydrate–Peptide Linkage of EPS

The EPS carbohydrate–peptide bond was analyzed using a β-elimination process. A solution of 2.0 mg/mL EPS was incubated with a mixture of 0.1 M NaOH and 1 M NaBH_4_ in a 45 °C water bath for 24 h. The sample was subsequently evaluated using a UV spectrophotometer (UV-2550, Shimadzu, Kyoto, Japan) with scanning wavelengths ranging from 190 to 400 nm. The obtained data were then compared with those of an untreated sample that had not undergone alkaline treatment.

### 2.7. Nuclear Magnetic Resonance (NMR) Spectroscopy Analysis of EPS

A solution containing 50 mg of EPS was prepared by dissolving EPS in 1 mL of D_2_O. This solution was then subjected to a comprehensive NMR analysis, specifically 1D and 2D NMR techniques (COSY and HSQC), utilizing an Avance III NMR spectrometer (Avance III, Bruker, Germany). The obtained NMR data were processed and analyzed using MestReNova software (MestReNova x64-14.2.1, Mestrelab Research, Santiago de Compostela, Spain). The obtained ^1^H and ^13^C spectra were meticulously examined, with chemical shifts within the NMR profile reported in units of parts per million (ppm). 

### 2.8. Scanning Electron Microscopy (SEM) Analysis of EPS

The morphological analysis of the EPS powder was performed using an SEM system, specifically the QUANTA FEG2 (FEI, Hillsboro, OR, USA) model, which was equipped with a Schottky emitter operating within a voltage range of −200 V to 30 kV. For the examination, the EPS sample was mounted on an aluminum support and coated with gold prior to SEM observation. Microscopic observations were recorded at two different magnifications, 100× and 1000×, to decipher the surface details of the samples.

### 2.9. Intrinsic Viscosity Measurement of EPS

The inherent viscosity characteristics of EPS were evaluated in a 0.1 M NaNO_3_ solution using an Ubbelohde capillary viscometer (Zhuoxiang Technology Co., Ltd., Hangzhou, China) with a diameter of 0.57 mm. This assessment was conducted in a water bath at 25 and 35 °C. The measurements were conducted at concentrations that maintained the relative viscosity, *η_r_*, between Equations (1) and (2), ensuring predominantly Newtonian flow behavior. The Huggins and Kraemer models were utilized for estimation to determine the [*η*] value.

(2) $$\frac{\eta_{sp}}{C}=\left[\eta \right]+K^{\prime }{\left[\eta \right]}^{2}C$$

(3) $$\frac{\left(\ln\eta_{r}\right)}{C}=\left[\eta \right]+K^{\prime \prime }{\left[\eta \right]}^{2}C$$

where *K*′ represents the Huggins parameter, whereas *K*″ denotes the Kraemer constant. The concentration of the solute is denoted as *C*. The relative viscosity, *ƞ_r_*, is defined as the ratio of the viscosity of the solution to that of the solvent (*ƞ_r_*/*ƞ_S_*). The specific viscosity, *ƞ_sp_* (*ƞ_r_ −* 1), is another important term in this context. The intrinsic viscosity was determined through an extrapolation process to reach a concentration of zero.

### 2.10. Thermal Properties of EPS

The thermal properties of the EPS were investigated using thermogravimetric analysis (TGA) and differential scanning calorimetry (DSC) with a thermal analysis instrument (Maia F3 200, Netzsch, Selb, Germany). A sample of approximately 10 mg of EPS was utilized, and TG–DSC curves were obtained over a temperature range of 40–800 °C under a nitrogen atmosphere with a temperature ramp rate of 10 °C/min.

### 2.11. Unraveling the Heavy Metal-Chelating Activity of EPS

An atomic absorption spectrometer (iCE 3500, Thermo Scientific, Waltham, MA, USA) was used to evaluate the metal-chelating activity of EPS according to the protocol outlined by Du et al. [27].

A mixture of 2 mL of the EPS solution with a concentration of 10 mg/mL was combined with 50 mL of a solution containing 10 mg/L Cu^2+^, Fe^2+^, and Zn^2+^ ions. The mixture was allowed to equilibrate at room temperature for 2 h, followed by a centrifugation step at 10,000 rpm for 10 min.

### 2.12. Measurement of the Antioxidant Capacity and Reducing Power of EPS

#### 2.12.1. 1,1-Diphenyl-2-picrylhydrazyl (DPPH) Radical Scavenging Activity

The antioxidant potential of EPS was evaluated using the DPPH method, according to protocol by Shen et al. [28]. In this procedure, 2.0 mL of EPS at various concentrations (ranging from 0 to 5.0 mg/mL in both crude and pure forms) was thoroughly mixed with 2.0 mL of a 0.2 mM DPPH–ethanol solution. After a 30-minute incubation period, the absorbance was measured at 517 nm. Ascorbic acid (Vc) was used as a positive control for comparison purposes. The results are presented as the percentage of scavenging efficacy.

#### 2.12.2. 2,2′-Azino-bis-(3-ethylbenzthiazoline-6-sulfonate) (ABTS) Radical Scavenging Activity

The capacity of EPS to decolorize ABTS radical cations was assessed using a previously documented method [29]. The absorbance was measured at a wavelength of 734 nm, with Vc used as a benchmark for comparison. The results are presented as the percentage of scavenging efficacy.

#### 2.12.3. Hydroxyl Radical Scavenging Activity

Hydroxyl radicals were generated using the method outlined by Liu et al. [30]. The absorbance of the final solution was measured at a wavelength of 510 nm, with Vc serving as a positive control. The results are presented as the percentage of scavenging efficiency.

#### 2.12.4. Reducing Power

The antioxidant capacity was assessed using the reducing power method according to the protocol outlined by Chen et al. [31], with the absorbance measured at a wavelength of 700 nm. Vc was used as the positive reference standard in this experiment. The reducing power was quantified in terms of its scavenging efficacy and was reported as a percentage.

#### 2.12.5. Oxidative Stress Induction by tBHP and Treatment of Cells with EPS

The protective effect of the EPS toward mouse insulinoma 6 (MIN-6) cells was tested using the CCK8 assay. The cells were cultured in RPMI 1640 medium (Keygen Biotech, Jiangshu, China) supplemented with 10% heat-inactivated fetal bovine serum (Clark, Arlington, VA, USA), 100 U/mL penicillin, and 100 U/mL streptomycin. Cultivation was conducted in a humidified atmosphere with 5% CO_2_ at 37 °C. Briefly, the 96-well plates were inoculated with a single-cell suspension of 1 × 10^5^ MIN-6 cells /mL in the logarithmic growth stage in a volume of 100 μL per well. MIN-6 cells were treated with 150 μM of tert-butyl hydroperoxide (tBHP) and co-cultured with different concentrations of EPS (50, 100, and 200 μg/mL). The control group and model group were treated with RPMI-1640 and 150 μM of tBHP, respectively. The plates were then cultured at 37 °C in an atmosphere containing 5% CO_2_ and maintained with saturated humidity for a period of 24 h. Following this incubation period, 10 μL of CCK8 (Cell Counting Kit-8) (GlpBio, Montclair, CA, USA) solution was added to each well. The plates were incubated at 37 °C for an additional duration of two hours. Finally, an enzyme-linked immunoassay (Infinite m plex, TECAN, Männedorf, Switzerland) was employed to measure the absorbance within each well at a wavelength of 450 nm. The percentage of cell viability for each tested sample was calculated using the following equation.

Cell viability (%) = A_EPS_/A × 100% (4)

where A_EPS_ is the absorbance value of the experimental group treated with EPS, and A is the absorbance value of the control group treated without EPS.

#### 2.12.6. ROS Assays

The MIN-6 cells were treated as described in Section 2.12.5. The levels of ROS was measured with the fluorogenic dye 2′, 7′-dichloro-fluorescin diacetate (DCFH-DA) staining using the ROS assay kit (Beyotime, Shanghai, China) coupled with an Infinite m plex (TECAN) plate reader, according to the manufacturer’s instructions. Briefly, following treatment, cells were centrifuged at 1000 rpm for 5 min, washed in 1 mL serum-free RPMI-1640 medium, then preincubated with DCFH-DA (10 μM) for 20 min at 37 °C. These were mixed upside down slightly every 5 min. After the extracellular dye was removed, the cells were washed three times with serum-free RPMI-1640 medium. Subsequently, fluorescence was measured at Ex/Em 488/525 nm by the plate reader. ROS levels were normalized to cell number.

#### 2.12.7. Statistical Analysis

GraphPad 8.0 software was used for statistical analysis of data. Results are presented as the mean ± SD of three independent experiments. Treatment effects were analyzed using the Student’s *t* test when comparing two variables, One-way ANOVA was performed and the Tukey’s multiple comparisons test was used to analyze inter-group differences. *p* < 0.05 was considered to be statistically significant.

## 3. Results

### 3.1. Analysis of the Fermentation Process for EPS Production

**Figure 1:**
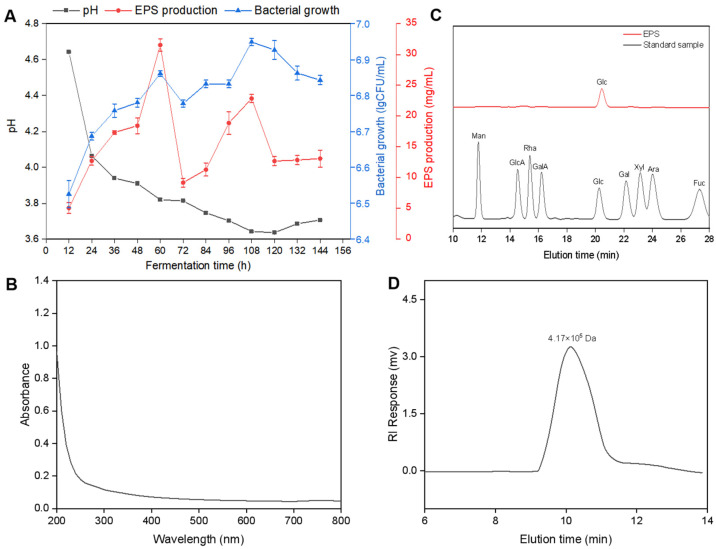
Fermentation course of *L. pseudomesenteroides* QY-1 in beet waste substrate (**A**) and the purity analysis by UV–Vis spectrum (**B**). Monosaccharide composition analysis by HPLC spectrum (**C**) and Mw analysis by HPSEC spectrum (**D**) of EPS.

The change in pH during fermentation is shown in Figure 1A. At 12 h of fermentation, the pH value of the fermentation system was 4.65. With increasing fermentation time, the pH of the fermentation system first rapidly decreased but then stabilized. After fermentation for 24 h, the pH of the fermentation system was 4.04, and the pH changed; this time point may be the stage at which a large number of bacteria replicate, using the substrate to produce a large amount of organic acid and lowering the pH of the fermentation system. After 108 h of fermentation, the fermentation system had the lowest pH of 3.65, which did not change significantly afterward. At the end of fermentation, the substrate utilization in the fermentation system may have been exhausted, and the strain could not continue to produce organic acids. The number of bacteria increased with increasing fermentation time. The increase of approximately 0.5 logarithmic units over the 24 h fermentation period demonstrated the robust multiplication capacity of the bacteria under these conditions and reached a maximum of 6.94 log CFUs/mL at 108 h of fermentation. The yield of EPS tended to first increase, then decrease, then increase, and was maintained at 13.10 mg/mL at the end of fermentation. In the MRS medium, the total yield of EPS from *L. Pseudomesenteroides* was 19.87 ± 0.45 g/L [24]. However, in the beet waste medium, the production of EPS by *L. pseudomesenteroides* reached a maximum value of 31.42 ± 1.07 g/L (Figure 1A). Zhao et al. [7] reported a maximum EPS yield of 7.75 g/L produced by *L. lactis* L2 after fermentation in MRS medium for 24 h at 30 °C. In a similar study, Zhou et al. [32] reported that the yield of EPS produced by the *L. pseudomesenteroides* XG5 strain inoculated in MRS medium supplemented with 12.5 g/L glucose was 35.5 mg/L. Furthermore, research conducted by Han et al. revealed that the exopolysaccharide generated by *L. mesenteroides* BD1710 in a tomato juice–sucrose mixture was dextran [33]. Wang et al. [3] reported a relatively high EPS yield of 3562.68 ± 81 mg/L produced by *Weissella cibaria* when maize straw was used as a carbon source. Liang et al. reported that when molasses was used as a substrate, the production of EPS by *Leuconostoc citreum* B-2 resulted in an increased yield of EPS to 48.45 ± 0.24 g/L [12]. Long et al. evaluated the EPS production capacity of *Leuconostoc suionicum* LSBM1 using sugar beet molasses and reported that the maximum EPS yield was 31.78 g/L when 300 g/L of beet molasses was used [34]. Moreover, the yields of EPS and CFU were 0.19 ± 0.02 g/g and 2.78 ± 0.13 CFU/g at 108 h, respectively. The results showed that *L. pseudomesenteroides* could use beet waste residue as a substrate to grow and produce EPS while reducing the pH of the fermentation system. Collectively, the utilization of low-cost substrates for the production of microbial exopolysaccharides represents a novel research direction. This approach provides a broader perspective on the high valorization potential of such substrates in academic research. EPS extracted from *L. pseudomesenteroides* in beet waste media presented the highest observed yield. Therefore, it was subjected to physical and chemical characterization, and its antioxidant potential was evaluated.

### 3.2. Homogeneity, Molecular Weight, and Monosaccharide Composition of EPS

UV–Vis spectroscopy revealed that the EPS had no characteristic absorption peak at 260 nm or 280 nm, indicating that the EPS was free of nucleic acid and protein contamination and had high purity (Figure 1B).

The results of the GC analysis are shown in Figure 1C. Compared with that of the standard sample, the characteristic peak of the EPS sample appeared only at 21.89 min, which is consistent with the characteristic peak of glucose. These results indicate that the EPS was a glucan composed of glucose and did not contain other monosaccharide components. Moreover, the EPSs synthesized from *Leuconostoc holzapfelii* KM01 and *L. mesenteroides* B3 were found to be composed of glucose [35,36], consistent with the results of this study. In contrast, the studies by Ziadi et al. [37] revealed that the isolates of *Lactococcus lactis* SLT10 produced EPSs composed of glucose, mannose, and rhamnose. Conversely, those originating from *L. mesenteroides* XR1 were primarily composed of glucose and mannose. This phenomenon demonstrates the complexity of exopolysaccharides produced by lactic acid bacteria. Further studies revealed that the polysaccharides found in *L. mesenteroides* are composed of mannose, arabinose, galactose, glucose, and fucose. When polysaccharides are isolated from bacterial sources, monosaccharides such as mannose, glucose, and galactose are commonly detected [38]. The composition of exopolysaccharides has a direct effect on their industrial applications, highlighting their diversity [11].

The results of the Mw determination of EPS are shown in Figure 1D. A distinct peak was observed in the EPS HPLC profiles at a retention time of 10.3 min. The deduced Mw of the EPS was 417 kDa, consistent with the previously reported Mw range of lactic acid bacteria EPS (10^4^–10^6^ Da), which was lower than that of the EPS generated by other *Leuconostoc* strains such as *L. citreum* BH10 (8990 kDa), *L. mesenteroides* RSG7 (5470 kDa), *L. mesenteroides* P35 (9900 kDa), and *L. mesenteroides* DRP105 (1.01 × 10^8^ Da) [39,40,41,42]. Previous studies have indicated that the biological effectiveness of EPS is influenced by its molecular weight. Ma et al. revealed that the low Mw of the glucan increases its solubility, thereby facilitating its use in enhancing stability and emulsifying properties [43]. Additionally, a decrease in the Mw of glucan enhances the immune response, diabetes-fighting properties, antiproliferative effects, and antioxidant and anti-inflammatory capabilities. It also improves the potential for cholic acid absorption in vitro [44,45].

### 3.3. Functional Group Composition of EPS

**Figure 2:**
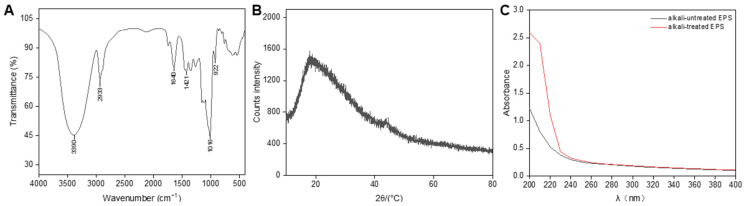
FTIR spectra (**A**), XRD spectra (**B**), and chain conformation analysis (**C**) of the EPS.

The polysaccharide composition demonstrated unique structures, as revealed by the FTIR analysis, which highlighted peaks for specific functional groups [46]. As shown in Figure 2A, the FTIR spectrum of the EPS functional groups exhibited typical polysaccharide characteristics. Notable absorption peaks included the vibrations of hydroxyl (OH) groups at 3390 cm^−1^, C–H stretching at 2933 cm^−1^, and carbonyl (C–O) groups at 1640 cm^−1^. The broad stretching from 910 to 1170 cm^−1^ was attributed to C–O–C and C–O linkages, indicating the presence of an α-pyranose structure within the polysaccharide [47]. The intense absorption peak between 1200 and 950 cm^−1^ serves as the fingerprint region for exopolysaccharides. Importantly, the peaks at 1016 cm^−1^ and 1020 cm^−1^ were previously associated with α (1→6) glycosidic bonds linked to α-D-glucose units [48,49], confirming their presence in the polysaccharide fractions designated EPS at 1016 cm^−1^.

### 3.4. XRD Spectroscopy Analysis of EPS

The use of XRD as an analytical technique is invaluable for determining the crystalline and amorphous features of materials. As shown in Figure 2B, the XRD profile of EPS revealed a distinct peak at approximately 20° on the 2θ scale, indicating a specific interplanar distance (d-spacing) of 4.86 A°. This discovery suggests that the EPS sample has a combination of crystalline and amorphous characteristics, resembling the properties of dextran derived from *Weissella confusa* H2 [50].

### 3.5. Chain Conformation of EPS

Two primary forms of glycosylation have been identified: N-glycosylation and O-glycosylation. N-glycosylation involves N-acetylglucosamine (GlcNAc) and mannose (Man), whereas O-glycosylation involves a broader range of linkages. An analysis of the monosaccharide composition revealed that EPS consisted solely of glucose excluding the presence of Man and GlcNAc, suggesting an O-type linkage in our sample [51]. β-Elimination was employed to further investigate the carbohydrate–peptide linkage. Under alkaline conditions, serine and threonine in O-glycosylations are converted to α-aminoacrylic acid and α-aminocrotonic acid, respectively, leading to increased absorbance at 240 nm [52]. A significant difference in absorbance at this wavelength was observed between the alkali-treated EPS and the untreated EPS, indicating the occurrence of a β-elimination reaction (Figure 2C). This evidence confirms the presence of an O-peptide bond in the polysaccharide, which aligns with findings from studies of *L. mesenteroides* DRP105 EPS [53].

### 3.6. NMR Spectroscopy Analysis

**Figure 3:**
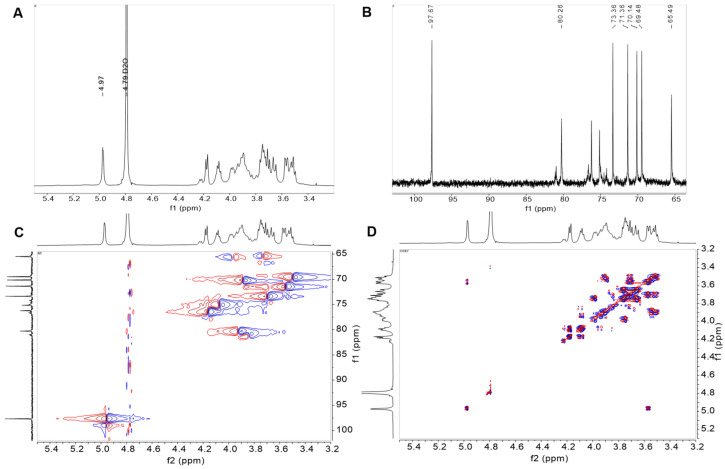
1D 1H (**A**), 13C (**B**) NMR spectra, and 2D COSY (**C**) and HSQC (**D**) NMR spectra of *L. pseudomesenteroides* EPS.

NMR spectroscopy is a well-established technique for analyzing the structural characteristics of EPS. The ^1^H NMR spectra provided detailed information on the glycosidic bond configurations including the anomeric proton regions (δ4.5–5.5 ppm) and the C2–C6 ring proton regions (δ3.1–4.5 ppm) (Figure 3A,B). The peak at 4.97 ppm indicated the presence of an α-(1,6)-linked D-glucopyranose moiety. In the ^13^C NMR spectrum, anomeric carbons typically exhibited chemical shifts between 95 and 110 ppm, whereas nonanomeric carbons resonated within the range of 50 to 85 ppm. The α-(1,6) linkage was confirmed by a signal at 97.67 ppm, and signals at 73.36, 71.36, 70.14, and 69.48 ppm represented the C3-, C2-, C5-, and C4-substituted glucose units, respectively. The main chain of EPS, which is interconnected via α-(1,6) linkages, was indicated by the C-6 carbon signal at 65.49 ppm. The absence of peaks from 101 to 105 ppm indicated the presence of α-configured glycosidic bonds in the EPS.

Insights into the configuration of EPS were obtained through 2D NMR analysis, specifically via HSQC spectroscopy. The presence of a single sugar moiety within the repeating unit was confirmed by the connection established by the proton signals in both the COSY and HSQC spectra of the EPS (Figure 3C,D). The anomeric region revealed a solitary cross peak, suggesting that a monosaccharide is a repeating unit with a carbon signal at δ97.67 ppm and a proton signal at δ4.97 ppm. The HSQC spectrum further revealed proton signals (4.97/97.67 for H1/C1, 3.56/71.36 for H2/C2, 3.67/73.36 for H3/C3, 3.53/69.48 for H4/C4, 3.86/70.14 for H5/C5, and 3.94/65.49 for H6/C6), confirming the presence of α−(1→6)-linked glucose units within the EPS glucose repeat structure. Integration of these NMR data revealed that the EPS structure was a linear α-(1,6)-linked dextran, similar to the *L. mesenteroides* TDS2-19 dextran but distinct from the *L. mesenteroides* DRP105 dextran, with α-(1→6) linkages in the main chain and α-(1→2) linkages at the branch point [42,54,55]. Notably, EPS solubility is structure dependent, as Maina observed that linear EPS has superior solubility, and branched structures can hinder solubility [56]. This finding suggests that EPS is more soluble than dextran, indicating its potential use in the food industry.

### 3.7. Morphological Properties of EPS

**Figure 4:**
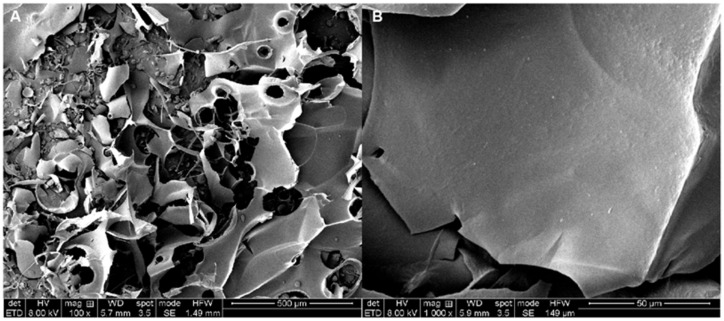
SEM showing the surface morphology of the EPS at various magnifications: at 100× (**A**) and 1000× (**B**).

SEM analysis contributed to the examination of the microstructural and surface features of EPS, thereby providing insights into its physical attributes. As shown in Figure 4, EPS exhibited an uneven, sleek, and interconnected web-like sheet structure. Upon closer inspection at a higher magnification, the EPS surface appeared even flatter and smoother, suggesting its capacity to enhance the rheological characteristics of food by increasing viscosity and water retention [57]. This irregular yet persistent mesh-like structure endows EPS with robust mechanical stability, while its smoothness enables the formation of flexible film materials. Similar EPS structures have been identified in *L. pseudomesenteroides* PC [58]. Numerous studies have validated the use of such EPSs in culinary applications as thickening and emulsifying agents because of their moisture retention capabilities and potential to generate plastic films [44].

### 3.8. Viscosity Specificity Analysis of EPS

**Figure 5:**
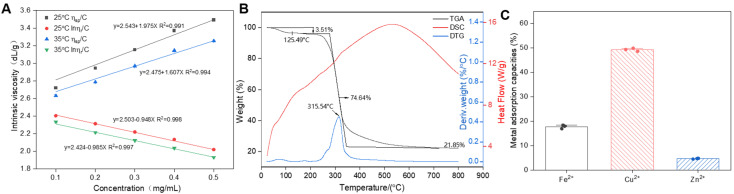
Analysis of viscosity characteristics (**A**), thermal stability (**B**), and heavy metal absorption characteristics (**C**) of *L*. *pseudomesenteroides* EPS.

Intrinsic viscosity is a fundamental characteristic of polysaccharide solutions that reflects the hydrodynamic volume occupied by a polymer and is therefore related to the dimensions and conformation of the polymer chains. In a specific and well-defined polysaccharide–solvent system at a constant temperature, the intrinsic viscosity of the solution remains constant. The Huggins and Kraemer Equations (1) and (2) were used to calculate the intrinsic viscosity of EPS by extrapolating the data to a zero concentration (Figure 5A). The intrinsic viscosity of EPS (2.5 dL/g) at 25 °C was greater than that at 35 °C (2.4 dL/g). The intrinsic viscosity of EPS decreased with increasing temperature, and its inherently low viscosity suggested a limited thickening capacity, indicating that a higher concentration may be needed to achieve the desired viscosity. Reports are available that provide detailed information on the intrinsic viscosity characteristics of EPS derived from different bacterial species. The EPS exhibited a higher intrinsic viscosity than *Lactobacillus casei* LC2W EPS (1.9 dL/g), but lower intrinsic viscosity than *Lactococcus lactis* EPS (19.6 dL/g) and *Bifidobacterium longum* EPS (32.0 dL/g) [59,60].

### 3.9. Thermodynamic Stability of EPS

An analysis of the thermal characteristics contributes to a more comprehensive understanding of the physiochemical attributes of EPS and expands its potential industrial applications. The TGA findings, as shown in Figure 5B, illustrate the degradation of EPS through three distinct phases. Initially, a 3.51% mass loss was observed between 30 °C and 100 °C, which was attributed to the abundant carboxyl groups in EPS. These groups facilitated the release of bound water at higher temperatures, resulting in a decrease in the EPS weight. Subsequently, a 74.64% mass reduction was detected from 250 °C to 350 °C due to high-temperature-induced depolymerization, leading to the breakdown of C–C and C=O bonds within the ring structure and significant weight loss through evaporation. The EPS weight stabilized between 350 °C and 700 °C. The DTG curve revealed an EPS degradation temperature (Td) of 315.54 °C, which surpassed that of *L. pseudomesenteroides* DRP-5 dextran (298.1 °C) [24]. Similar findings have shown that an EPS produced by *L. citreum* B-2 remained stable at temperatures up to 313 °C [61]. According to Bomfim et al., the similar thermal stability and degradation behavior are likely attributed to the comparable carbohydrate compositions of these polysaccharides [62]. The stability of the EPS weight at elevated temperatures highlights its complex molecular structure, monosaccharide constitution, and molecular weight. This higher degradation temperature indicates superior thermal stability, positioning the isolated EPS favorably for use in food chemistry applications. The DSC profile showed a clear endothermic melting peak at 514.27 °C upon initial heating, which was associated with water vaporization and the melting of long aliphatic side chain-formed crystalline structures within the EPS molecules [63]. Overall, these results from the DSC, TGA, and DTG analyses confirm that EPS is suitable for diverse applications across various industries including the food chemistry sector, pharmaceuticals, and the chemical industry.

### 3.10. Heavy Metal-Chelating Activity of EPS

Certain EPSs possess a variety of functional groups including hydroxyl, ketonic, alcoholic, amine, thiol, and carboxylic groups. These diverse functional groups endow EPS with multiple negative charges, which facilitate the extraction of positively charged heavy metal ions from aqueous solutions. This finding highlights EPS as a potential metal-chelating agent. As shown in Figure 5C, EPS demonstrated high efficacy in removing Cu^2+^ from solutions, with an adsorption capacity of 49.29%. However, its adsorption rates for Fe^2+^ and Zn^2+^ (17.70% and 4.60%, respectively) were lower than those reported for *L. mesenteroides* HDE−8 EPS (Fe^2+^ at 87.56%, Zn^2+^ at 90.69%, and Cu^2+^ at 82.84%) [6]. The EPS displayed commendable heavy metal adsorption capabilities in water, potentially mitigating the harm of heavy metals to aquaculture and soil while fostering sustainable growth in aquaculture and vegetable farming.

### 3.11. Antioxidant Properties of EPS

#### 3.11.1. ABTS Radical Scavenging Activity

**Figure 6:**
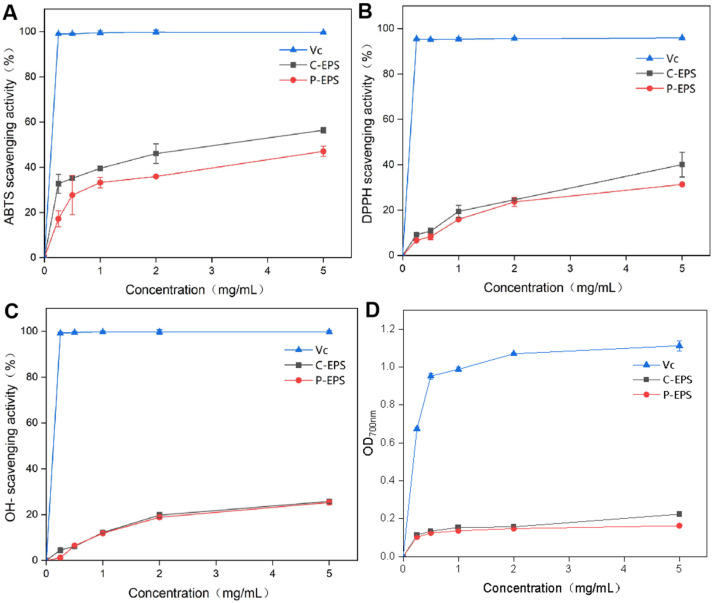
Antioxidant analysis of *L. pseudomesenteroides* EPS. (**A**) ABTS radical scavenging activity. (**B**) DPPH radical scavenging activity. (**C**) OH radical scavenging activity. (**D**) Reducing power assay.

The ABTS scavenging ability of EPS is shown in Figure 6A. With increasing concentration, the scavenging ability gradually increased. At 0.2 mg/mL, the ABTS scavenging ability of Vc reached more than 99%, while the ABTS scavenging ability of pure EPS was 17.2%. At a concentration of 5 mg/mL, the ABTS scavenging ability of pure EPS was 47.1%. In addition, the ABTS scavenging ability of the crude EPS was significantly greater than that of the pure EPS. At a concentration of 5 mg/mL, the ABTS scavenging ability of the crude EPS was 56.4%. At a concentration of 8 mg/mL, the EPS from *L. plantarum* KX041 demonstrated an impressive radical scavenging efficacy of approximately 80% for the ABTS radical [64]. In contrast, at the same concentration, the EPS from *Bacillus velezensis* SN-1 exhibited a scavenging effect of approximately 60% for the ABTS radical [65].

#### 3.11.2. DPPH Radical Scavenging Activity

Similar to the ABTS scavenging ability, the DPPH scavenging ability of EPS gradually increased with increasing concentration (Figure 6B). At a concentration of 0.2 mg/mL, the DPPH scavenging ability of Vc reached 95.5%, and that of pure EPS was 6.7%. At a concentration of 5 mg/mL, the DPPH scavenging ability of pure EPS was 31.4%, and that of crude EPS was 40.1%. No significant difference in DPPH scavenging ability was observed between the crude EPS and pure EPS at other concentrations. Within the scope of this study, the capacity of EPS to neutralize DPPH radicals exhibited a direct relationship with the EPS concentration. Consequently, higher levels of EPS led to an increase in DPPH scavenging activity. Previous research has shown that at a dosage of 4.0 mg, EPS (LPC-1) was 52.23% effective at scavenging DPPH radicals, whereas ascorbic acid was more effective at 88.60% [2,66].

#### 3.11.3. OH Radical Scavenging Activity

Among all of the oxygen-derived free radicals, highly reactive hydroxyl radicals engage in multiple interactions with diverse biological macromolecules within living cells. These reactions may involve triggering lipid peroxidation processes and leading to DNA impairment. Consequently, neutralizing these hydroxyl radicals constitutes a vital strategy for safeguarding cells and tissues from oxidative stress-induced damage [67]. These findings highlight the potential significance of EPSs in various biomedical applications. The OH scavenging ability of the sample is shown in Figure 6C. When the concentration was 0.2 mg/mL, the OH removal capacity of Vc reached 99%. No significant difference in OH scavenging ability was observed between crude EPS and pure EPS, and the scavenging ability gradually increased with increasing EPS concentration. At a concentration of 5 mg/mL, the OH scavenging ability of EPS was 25.2%. In a study conducted by Wang et al., the antioxidant properties of EPS derived from *Lactobacillus fermentum* were assessed [68]. The authors reported a hydroxyl radical scavenging effect of 61.67% at a concentration of 4 mg/mL, which is slightly greater than the results from the current investigation. These findings suggest that the EPS under investigation has a significant ability to neutralize hydroxyl radicals. Previous research has indicated that the ability of EPS to scavenge hydroxyl radicals is achieved through the inhibition of hydroxyl radical production by binding to Fe^2+^ or Cu^2+^, which are potent pro-oxidants involved in oxidative catalysis [54,56].

#### 3.11.4. Reducing Power Assay

The total reducing power is a significant parameter for evaluating the antioxidant capacity of a given sample [53]. The total reducing power of the sample is shown in Figure 6D, and the total reducing power of the sample increased with increasing concentration. When the concentration was 0.2 mg/mL, the OD_700nm_ value of the Vc sample was 0.67, while the OD_700nm_ value of EPS was approximately 0.11, and no significant difference was observed between the pure EPS and crude EPS. When the concentration was 2 mg/mL, the OD_700nm_ value of the Vc sample was 1.07, the OD_700nm_ value of the EPS was approximately 0.15, and then remained stable, which was lower than the reducing power of EPS-3 from *Weissella cibaria* SJ14 (0.56 at 5.0 g/L) [69].

#### 3.11.5. Protective Effects of EPS on tBHP-Induced Oxidative Damage in MIN-6 Cells

**Figure 7:**
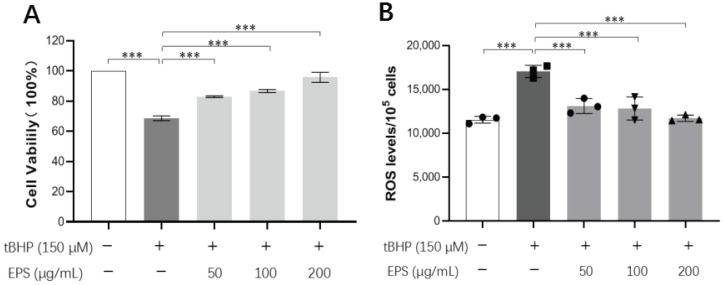
Intracellular antioxidant activity of EPS. (**A**) The protective effect of EPS on the viability of MIN-6 cells exposed to tBHP. (**B**) The effect of EPS on intracellular ROS level in tBHP-induced MIN-6 cells. The cells were treated with 150 μM of tBHP and co-cultured with different concentrations of EPS (50, 100, and 200 μg/mL). The control group and model group were treated with RPMI-1640 and 150 μM of tBHP, respectively. All data are expressed as mean ± SEM (n = 3). Statistical significance is represented as follows: *** *p* < 0.001.

Pancreatic beta cells are highly vulnerable to stress due to their weak antioxidant capacity [70]. Excessive intracellular ROS could damage the structure and function, leading to oxidative damage. Effective antioxidants can mitigate oxidative stress-induced damage in pancreatic β-cells. Based on the antioxidant activity exhibited by EPS in vitro, we investigated the effect of EPS on tBHP-induced oxidative damage in MIN-6 cells to further explore its antioxidant activity. However, EPS demonstrated protective effects against tBHP-induced oxidative damage in MIN-6 cells. As shown in Figure 7A, the viability of MIN-6 cells incubated with 150 μM tBHP was significantly lower than that of the control group. Notably, these effects were reversed in a dose-dependent manner when the cells were treated with EPSs (50, 100, and 200 μg/mL). The EPSs significantly increased the cell viability compared to the tBHP-induced group. Similarly, Guzmán et al. [71] reported that lupin γ-conglutin protects against cell death induced by oxidative stress, with γ-conglutin providing partial protection to MIN-6 cells against H_2_O_2_-induced death. Liu et al. [72] found that EPSs from *Lactobacillus fermentum* LFQ153 had a protective effect against oxidative damage in RAW264. 7 macrophages. Since the protective effect of EPS on the viability was obvious in MIN-6 cells exposed to oxidative stress, we proceeded to measure the ROS content in these cells. As anticipated, we observed significantly elevated intracellular ROS levels following tBHP-induced stress in MIN-6 cells (Figure 7B). MIN-6 cells treated with EPS exhibited a marked reduction in intracellular ROS levels compared to the tBHP-induced group. Remarkably, this effect was attributed to EPS treatment. The ROS levels in the MIN-6 cells treated with EPS at a concentration of 200 μg/mL were comparable to those in the control group of non-exposed cells. These findings suggest that EPS can protect MIN-6 cells from tBHP-induced oxidative damage, most likely by suppressing intracellular ROS production. In a related study, Huang et al. [73] reported that acidic EPS (EPS-LP2) isolated from *Lactiplantibacillus plantarum* DMDL 9010. EPS-LP2 protected RAW264.7 cells from oxidative injury by reducing ROS generation.

## 4. Conclusions

In this study, the EPS samples produced by *L. pseudomesenteroides* were isolated and purified, and the structure and properties of the EPS were characterized. The EPS was identified as a homopolysaccharide with a glucose-based repetitive unit. Spectroscopic analyses using FTIR and NMR revealed the presence of carbohydrate-linked functional groups, specifically carboxyl and hydroxyl moieties. Microscopic observations revealed a highly porous architecture, resulting in film-like properties of the EPS. The XRD data suggested a semicrystalline structure. Moreover, the EPS demonstrated good thermal stability, specific viscosity, and oxidation resistance. Importantly, the EPS exhibited significant protective effects on tBHP-induced intracellular oxidative stress. These findings suggest the potential application of the EPS from *L. pseudomesenteroides* as an antioxidant agent in functional food products and pharmaceutical applications.

## Data Availability

Data are contained within the article.
